# Development and Characterization of EGCG-Loaded TPGS/Poloxamer 407 Micelles with Evaluation of In Vitro Drug Release and In Vivo Pharmacokinetics and Tolerability Observations Following Oral Administration

**DOI:** 10.3390/pharmaceutics17111441

**Published:** 2025-11-07

**Authors:** Chee Ning Wong, Kai Bin Liew, Yang Mooi Lim, Yik-Ling Chew, Ang-Lim Chua, Shi-Bing Yang, Siew-Keah Lee

**Affiliations:** 1 M. Kandiah Faculty of Medicine and Health Sciences, Universiti Tunku Abdul Rahman, Kajang 43000, Malaysia; 2 Faculty of Pharmacy, University of Cyberjaya, Cyberjaya 63000, Malaysia; 3 Faculty of Pharmaceutical Sciences, UCSI University, Wilayah Persekutuan Kuala Lumpur 56000, Malaysia; 4 Faculty of Medicine, Universiti Teknologi MARA, Sungai Buloh 47000, Malaysia; 5 Institute of Biomedical Sciences, Academia Sinica, Taipei 115201, Taiwan

**Keywords:** catechin, drug release, EGCG, phytochemical-based nanomedicine, pharmacokinetics, micelle, poloxamer, TPGS

## Abstract

**Background**: Epigallocatechin-3-gallate (EGCG), a potent green tea polyphenol, possesses significant therapeutic potential, but its clinical application is limited by poor gastrointestinal stability and low oral bioavailability. To address this, a novel herbal nanomedicine-based delivery system was developed utilizing D-α-tocopheryl polyethylene glycol succinate (TPGS) and Poloxamer 407. **Objectives**: This study aims to develop and characterize EGCG-loaded TPGS/Poloxamer 407 micelles, evaluating their physicochemical properties, storage stability, in vitro drug release profile, in vivo oral bioavailability, and preliminary tolerability observation. **Methods**: The micelles were prepared using the film hydration method followed by lyophilization. **Results**: The optimized 2:2 TPGS-to-poloxamer 407 weight ratio yielded EGCG-loaded micelles, displaying a mean particle size of 15.4 nm, a polydispersity index (PDI) of 0.16, a zeta potential of −17.7 mV, an encapsulation efficiency of 82.7%, and a drug loading capacity of 7.6%. The critical micelle concentration (CMC) was determined to be 0.00125% *w*/*v*. Transmission electron microscopy (TEM) confirmed the micelles’ uniform spherical morphology. In vitro release studies demonstrated a sustained release profile in both simulated gastric and intestinal fluids. EGCG formulation remained stable for at least six months when stored at 4 °C. No adverse clinical signs were noted during the 28-day tolerability observation. In vivo pharmacokinetic evaluation in mice revealed a significant elevation in oral bioavailability, achieving a 2.27-fold increase in area under the curve (AUC) and a 1.8-fold increase in peak plasma concentration (Cmax) compared to free EGCG. **Conclusions**: Collectively, these findings underscore the potential of the TPGS/poloxamer 407-based micelle system as a promising oral delivery platform for EGCG, enhancing its stability and pharmacokinetic performance.

## 1. Introduction

Epigallocatechin-3-gallate (EGCG), a potent polyphenol, is the most abundant catechin in green tea, and has been extensively studied for its various pharmacological properties. Preclinical and early clinical investigations have demonstrated its efficacy in ameliorating several chronic pathological conditions especially those implicated in vascular dysfunction, type 2 diabetes mellitus (T2DM), and hypertension [1,2,3,4,5]. Extensive studies have shown that these therapeutic benefits are largely attributed to EGCG’s potent antioxidant, anti-inflammatory, and vaso-protective actions [6,7,8].

However, despite its promising pharmacodynamic profile, the clinical translation of EGCG is significantly hindered by its poor biopharmaceutical characteristics. EGCG is known to be unstable in gastrointestinal (GI) fluids, undergoes extensive first-pass metabolism, and has low oral bioavailability. Upon oral ingestion, EGCG is rapidly metabolized via hydrolysis, methylation, sulfation, and glucuronidation [9,10,11,12]. These metabolic conversions produce derivatives with altered biological activity [13,14]. Moreover, the compound is chemically unstable under alkaline intestinal conditions due to autooxidation of its pyrogallol ring [15,16], which leads to the generation of hydrogen peroxide and compromises its structural integrity [17,18]. In addition, EGCG’s passive absorption is limited by efflux transporters such as multidrug resistance protein 2 (MRP2), which actively extrudes it from enterocytes, further diminishing its systemic availability [13,19]. Moreover, early direct contact of EGCG with the GI lining has been associated with drug–drug interactions and altered or unfavorable drug metabolism [20,21].

These pharmacokinetic barriers result in sub-therapeutic plasma concentrations and inconsistent clinical outcomes, thereby restricting the broader integration of phytoconstituent into evidence-based therapeutic regimens. To address these challenges, the field of phytopharmaceuticals has increasingly turned to nanotechnology-based delivery systems. Herbal nanomedicines utilizing nano-carriers such as liposomes, micelles, nano-emulsions, and polymeric nanoparticles have demonstrated the capacity to enhance solubility [22], protect labile compounds from enzymatic and oxidative degradation [23], improve permeability [24], and prolong systemic circulation time with reduced toxicity [25], offering innovative solutions to the long-standing challenges associated with conventional phytochemical formulations. Another critical benefit of nanotechnology is the protection of labile phytochemical compounds from degradation caused by environmental factors such as heat, light, and oxidative stress [26]. Nano-formulations stabilize these bioactives, enhancing their shelf life and maintaining their biological activity under physiological conditions.

Various strategies have been employed to enhance EGCG’s bioavailability and therapeutic effects, including nano-encapsulation [27,28], liposomal and phytosome delivery [29,30], and co-administration with metabolic enzyme inhibitors such as piperine and curcumin [31,32]. Structural modifications and synthetic analogs of EGCG have also been explored to improve stability under physiological conditions [13]. However, many of these approaches provide only partial protection, display inconsistent release kinetics, or introduce complexities in formulation and scalability. Furthermore, most studies have inadequately characterized the complete pharmacokinetic profile of EGCG-loaded nanoparticles, leaving critical gaps in understanding their absorption, distribution, metabolism, excretion, and potential toxicity [12].

In this context, novel polymeric nanoparticle-based delivery systems employing D-α-tocopheryl polyethylene glycol succinate (TPGS) and Poloxamer 407 amphiphilic micelles have been proposed to more effectively address these challenges. TPGS, a water-soluble derivative of vitamin E, serves as both a stabilizer and an inhibitor of efflux transporters such as MRP2, thereby enhancing intestinal absorption and cellular uptake [33]. Poloxamer 407, a non-ionic triblock copolymer, improves drug permeability by reducing mucosal viscosity and enhancing transcellular transport [34]. Both excipients are biocompatible and widely used in pharmaceutical formulations [33,35]. These amphiphilic components form stable micelles capable of encapsulating EGCG, protecting it from degradation, and facilitating sustained release. The micellar system is anticipated to improve the oral bioavailability of EGCG by circumventing its metabolic and absorption-related limitations. Therefore, this study aims to develop and characterize EGCG-loaded TPGS/Poloxamer 407 micelles and to evaluate their physicochemical properties, storage stability, in vitro drug release profile, in vivo oral bioavailability, and tolerability.

## 2. Materials and Methods

### 2.1. Materials

EGCG (>94% purity), vitamin E TPGS, poloxamer 407 were procured from Taiyo Kagaku (Mie, Japan), MedChem Express (Monmouth Junction, NJ, USA) and Sigma-Aldrich (St. Louis, MO, USA), respectively.

### 2.2. Preparation of EGCG-Loaded TPGS/Poloxamer 407 Micelle

EGCG-loaded TPGS/Poloxamer 407 micelle was prepared using the thin-film hydration method [36]. EGCG was dissolved with TPGS and Poloxamer 407 in absolute (99.7%) ethanol, and the mixture was stirred until a homogenous solution formed. The ethanol was then evaporated using a rotary evaporator and further dried under vacuum overnight to produce a thin film. Approximately 30 mL of water was added to rehydrate the thin film while stirring and heating for one hour, resulting in a micellar solution. This solution was centrifuged at 5000 rpm for 30 min and subsequently filtered through a 0.22 µm syringe filter. 3 mL of the final micellar solution was transferred into a sterile 10 mL glass vial and pre-frozen at −80 °C overnight. The freeze-drying process was conducted using a freeze dryer (Scanvac, LaboGene, Lillerød, Denmark). Once the condenser’s temperature reached below −90 °C, the frozen micellar solution was transferred into the drying chamber. A vacuum was then applied to the drying chamber and maintained for approximately three days. Upon completion of the freeze-drying cycle, the vial was removed, sealed tightly with screw cap, and stored at 4 °C.

### 2.3. Optimization of TPGS:Poloxamer 407 Ratio

The EGCG-loaded micelle was prepared as described in Section 2.2, with the amount of EGCG kept constant while the weight ratio of TPGS to Poloxamer 407 was systematically varied across three levels: 3:1, 2:2, and 1:3. Each micellar solution was then comprehensively evaluated for critical quality attributes, including particle size, PDI, zeta potential, encapsulation efficiency and drug loading capacity.

As detailed in Section 3.1, the formulation with a TPGS:Poloxamer 407 weight ratio of 2:2 demonstrates optimal characteristics across all evaluated parameters. Based on this comprehensive evaluation, the 2:2 TPGS:Poloxamer 407 ratio is selected as the optimized EGCG-loaded micellar formulation for all subsequent studies and simply referred as ‘EGCG micelle’ throughout this manuscript.

### 2.4. Critical Micelle Concentration (CMC)

The critical micelle concentration (CMC) of the TPGS/Poloxamer 407 mixture (2:2) was determined using iodine probe method [37,38], with slight modifications. A potassium iodide (KI)/iodine (I_2_) standard solution was prepared by dissolving 0.1 g of iodine and 0.2 g of potassium iodide in 10 mL of distilled water. 1% (*w*/*v*) TPGS/Poloxamer 407 stock solution was prepared and serially diluted to concentrations ranging from 0.000001% to 0.1%. To each diluted mixture, 25 µL of the KI/I_2_ standard solution was added, followed by 12 h of incubation in the dark at room temperature. Absorbance was measured at 366 nm using a UV-Vis spectrophotometer (Cary 100, Agilent, CA, USA). The CMC value was determined from the polymer concentration at which a sharp increase in absorbance was observed in the plot of absorbance versus logarithmic of TPGS/Poloxamer 407 mixture concentration.

### 2.5. Particle Size, Polydispersity Index and Zeta Potential

The particle size and polydispersity index (PDI) of the micelle were measured using dynamic light scattering (DLS) (BeNano 180 Zeta Pro, Bettersize, Liaoning, China). Freeze-dried micellar powder was dissolved in 5 mL of ultra-pure water and sonicated for 5 min to ensure complete dissolution. The analysis was conducted at 25 °C using a 50 mW solid-state laser (671 nm) with a scattering angle of 173°. The zeta potential of the micelles was also measured (BeNano 180 Zeta Pro, Bettersize, Liaoning, China). Parameters were measured in six replicates and presented as mean ± SEM.

### 2.6. Encapsulation Efficiency and Drug Loading Capacity

1 mg/mL of freshly prepared micellar solution was diluted 100 times with distilled water and then analyzed. The absorbance of the solution was measured at 274 nm using a UV-Vis spectrophotometer (Cary 100, Agilent, CA, USA. This assay was carried out in triplicate, and results are presented as mean ± SEM.

The equations of calculating encapsulation efficiency (EE) and drug loading capacity (DLC) in percentage are shown in (1) and (2):

(1) $$Encapsulation\;efficiency\left(\%\right)=\frac{Amount\;of\;EGCG\;loaded}{Initial\;amount\;of\;EGCG\;added}\times 100\%$$

(2) $$Drug\;Loading\;capacity\left(\%\right)=\frac{Amount\;of\;EGCG\;loaded}{\left(Amount\;of\;EGCG+TPGS+poloxamer\;407\right)}\times 100\%$$

### 2.7. Fourier-Transform Infrared (FTIR) Spectroscopy

The functional group identification and profiling of EGCG, TPGS, poloxamer 407 and EGCG-loaded micelle were conducted using Fourier-transform infrared (FTIR) spectrometer (Nicolet iS10, Thermo Fisher Scientific, MA, USA). The analysis utilized the Attenuated Total Reflectance (ATR) sampling technique, scanning sample across mid-infrared region from 4000 to 400 cm^−1^.

### 2.8. X-Ray Diffractometry (XRD)

The crystalline structures of EGCG, TPGS, poloxamer 407, and EGCG-loaded micelles were analyzed using an X-ray diffractometer (LabX XRD-6000, Shimadzu, Kyoto, Japan). The measurement was conducted over a scanning range of 5° to 70° (2θ) with a scanning speed of 2°/min.

### 2.9. Transmission Electron Microscopy (TEM)

The morphology of EGCG-loaded micelle was studied with a transmission electron microscope (Tecnai G2 20 S-Twin, FEI, OR, USA). A small amount of micellar powder was placed in a microcentrifuge tube and added with 10 mL of deionized water. The mixture was sonicated for 5 min, and a drop of the resulting solution was placed onto a copper grid. After allowing the sample to dry for 1 h, the surface morphology of the micelle was visualized.

### 2.10. Storage Stability

The freeze-dried EGCG-loaded micellar powder was divided into two portions and stored in the dark at controlled temperatures of 4 °C and 25 °C, respectively. The particle size, PDI, and zeta potential of the micellar powders were evaluated at 0.5, 1, 3 and 6 months, following the procedure described in Section 2.5.

### 2.11. In Vitro Drug Release Study

The drug release profiles of free EGCG and EGCG-loaded micelles were evaluated using the dialysis bag method in enzyme-free simulated gastric fluid (SGF, pH 1.2 ± 0.1) and simulated intestinal fluid (SIF, pH 6.8 ± 0.1) [36]. SGF was prepared by dissolving 2 g of sodium chloride and 7 mL of hydrochloric acid in 1000 mL of distilled water, while SIF was prepared by dissolving 6.8 g of potassium phosphate monobasic and 0.616 g of sodium hydroxide in 1000 mL of distilled water. The saturation solubility of EGCG in SGF and SIF was assessed by adding an excess amount of EGCG to the medium and shaking for 24 h at 37 °C to ensure equilibrium. The mixture was filtered and the EGCG concentration in the solution was measured using UV-Vis. The saturation solubility of EGCG in SGF and SIF is 63 and 87 mg/mL, respectively. Approximately 5 mg of EGCG and 5 mg EGCG equivalent of EGCG-loaded micelle were dissolved in 5 mL of distilled water, loaded into separate dialysis bags (12–14 kDa), and sealed. The dialysis bags were submerged in 100 mL of SGF or SIF and incubated at 37 °C with shaking at 100 rpm. The sink conditions were maintained throughout the experiment. At designated time points, 10 mL of the release medium was withdrawn and replaced with fresh medium to maintain a constant volume. The absorbance of the collected sample was measured at 274 nm using a UV-Vis spectrophotometer (UV-1800, Shimadzu, Kyoto, Japan). This assay was carried out in triplicate, and results are presented as mean ± SEM.

Cumulative drug release percentage at each time point was calculated using Equation (3).

(3) $$Cumulative\;EGCG\;release\left(\%\right)=\frac{Cumulative\;amount\;of\;EGCG\;released}{Amount\;of\;EGCG\;loaded\;in\;micelle}\times 100\%$$

### 2.12. Experimental Animals

The experimental protocols were approved by the Institutional Animal Care and Use Committee of Academia Sinica, Taiwan (Protocol Number: 24-08-2258). Animals were bred and maintained in-house at the Animal Facilities, Institute of Biomedical Sciences, Academia Sinica, Taiwan. The animals were housed on a standard 12 h light/dark cycle at 20–22 °C with ad libitum access to regular chow and distilled water.

### 2.13. Preliminary Tolerability Observations

A preliminary tolerability observation was conducted to assess the safety of EGCG micelles. Male C57BL/6 mice (29–34 g) were randomly allocated into three groups (*n* = 2 per group). For 28 consecutive days, mice received daily oral gavage administrations of distilled water, 100 mg/kg body weight EGCG, or 100 mg/kg body weight EGCG micelle. 100 mg/kg body weight dosage has been chosen as it is both pharmacologically relevant and well within a safe range for this study [1,39,40,41]. Body weight was monitored daily. To identify potential adverse events, cage-side observations for behavioral changes and specific signs of EGCG and EGCG micelles (e.g., inactivity, aggression, porphyrin staining, eye or skin inflammation, piloerection, reduced appetite, diarrhea, breathing difficulties, and mortality) were performed at 15 min and 4 h post-administration throughout the experimental period (Table 1) [1].

On day 29, mice were humanely sacrificed via cervical dislocation. Blood was collected from the tail vein into plain blood collection tubes (Microvette^®^ CB 300, Sarstedt, Nümbrecht, Germany) and then centrifuged at 3000× *g* for 10 min at 4 °C to yield serum for subsequent biochemical analysis, including alanine aminotransferase (ALT), aspartate aminotransferase (AST), urea, and creatinine.

After sacrifice, mice underwent dissection for a thorough examination of their internal organs. Thoracic organs (heart and lungs) and abdominal organs (liver, stomach, spleen, small intestine, large intestine, kidneys) were visually inspected for any gross lesions, alterations in size, or changes in external appearance. Subsequently, these organs were excised, blotted dry, and weighed. Relative organ weight was calculated using Equation (4):

(4) $$Relative\;organ\;weight=\frac{Organ\;weight\left(g\right)}{Body\;weight\left(g\right)}\times 100$$

### 2.14. In Vivo Pharmacokinetic Study

Male C57BL/6 mice (23–28 g) were randomly divided into two groups: EGCG (*n* = 7) and EGCG micelle (*n* = 6). They were orally administered a single dose of either EGCG (100 mg/kg body weight) or EGCG micelle (100 mg/kg body weight). A dosage of 100 mg/kg was selected, as it is well-documented in previous pharmacokinetic studies of EGCG [42,43] and has been demonstrated to be safe at this level [1,41]. Blood samples were collected at baseline (pre-administration) and at 1, 2, 4, 6, 8, 12, and 24 h post-administration via the tail snip method. A small portion of the tail tip was carefully excised using a sterile razor blade, and blood was collected into EDTA-coated blood collection tubes (Microvette^®^ CB 300, Sarstedt). Samples were centrifuged at 3000× *g* for 10 min, and the collected plasma was mixed with ascorbic acid-EDTA solution and immediately stored at −80 °C until further analysis.

The sample preparation for EGCG quantification in plasma samples was performed [44]. The plasma samples were mixed with ethyl gallate and extracted with ethyl acetate. The mixtures were vortexed for 15 min and centrifuged at 1900× *g* for 20 min at 4 °C. The supernatant was transferred to a clean microcentrifuge tube, and the extraction process was repeated for the residual pellet. The combined organic layers were evaporated to dryness using a centrifugal vacuum concentrator (miVac, Genevac, Ipswich, England). The dried residue was reconstituted in 0.3% acetic acid in 15% acetonitrile, vortexed for 15 min, and centrifuged at 16,200× *g* for 10 min at 4 °C. The supernatant was collected and stored at 4 °C before analysis.

Plasma EGCG concentrations were analyzed using an ultra-performance liquid chromatography–tandem mass spectrometry (UPLC-MS/MS) system (ACQUITY UPLC, Waters, Milford, MA, USA) equipped with an ACQUITY UPLC HSS T3 column (1.8 μm, 2.1 × 100 mm, Waters, MA, USA). The mobile phases consisted of water with 0.01% formic acid (solvent A) and acetonitrile with 0.01% formic acid (solvent B), delivered at a flow rate of 0.4 mL/min. The injection volume was 5 µL, and the column temperature was maintained at 40 °C. The UPLC system was interfaced with a Waters Xevo TQ-XS triple quadrupole mass spectrometer. Characteristic transitions were monitored for ethyl gallate (*m*/*z* 197 > 124) as the internal standard and EGCG (*m*/*z* 457 > 169).

### 2.15. Statistical Analysis

Data are presented as mean ± standard error of the mean (SEM). Statistical analyses were conducted using IBM SPSS Statistics version 22 (IBM Corp., Armonk, NY, USA). Differences between two groups were assessed using the independent samples *t*-test, while comparisons among multiple groups were analyzed using one-way ANOVA followed by Tukey’s post hoc test. A *p*-value of <0.05 was considered statistically significant.

## 3. Results and Discussion

### 3.1. Optimization of Ratio of TPGS:Poloxamer 407

**Figure 1:**
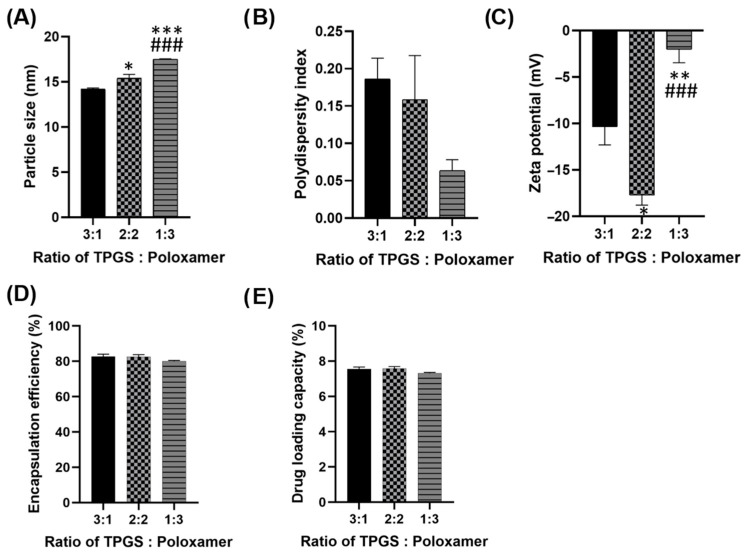
(**A**) Particle size (**B**) Polydispersity index (PDI) (**C**) Zeta potential (**D**) Encapsulation efficiency and (**E**) Drug loading capacity of different ratios of TPGS: Poloxamer 407. Parameters were measured in six replicates and presented as mean ± SEM. * *p* < 0.05 compared to ratio 3:1. ** *p* < 0.01 compared to ratio 3.1. *** *p* < 0.001 compared to ratio 3.1. ### *p* < 0.001 compared to ratio 2:2.

The ratio of TPGS to poloxamer 407 in the micellar formulation is optimized, and the composition demonstrating the best performance across key parameters—particle size, PDI, zeta potential, encapsulation efficiency and drug loading capacity—is selected for further preparation and evaluation. All three formulations exhibit particle size within 14–18 nm (Figure 1A), suggesting their potential for prolonged systemic circulation by evading the reticuloendothelial system (RES) recognition and destruction. Although the 2:2 formulation has a slightly larger particle size (15.4 nm) compared to the 3:1 ratio, it remains within the optimal nanometric range for efficient drug delivery, ensuring a high surface area and effective cellular uptake.

In Figure 1B, all three formulations achieved desirable PDI values (0.06–0.19), reflecting their high homogeneity. The 2:2 formulation demonstrates a PDI of 0.16, indicating a uniform particle size distribution. Although the 1:3 ratio exhibits a slightly lower PDI, the difference is negligible, and the 2:2 ratio still ensures homogeneity and consistency in the formulation. Among the three formulations, the 2:2 ratio displays the most favorable zeta potential (−17.7 mV), contributing to superior colloidal stability (Figure 1C). A higher absolute zeta potential reduces the likelihood of particle aggregation, enhancing storage stability and performance in biological system. The 2:2 formulation achieves a high encapsulation efficiency (EE) of 82.7%, comparable to the 3:1 ratio and superior to the 1:3 ratio (Figure 1D). A high EE ensures that more of the drug is encapsulated, enhancing therapeutic efficacy. The 2:2 formulation demonstrates a slightly better drug loading capacity (DLC), 7.6% compared to the other ratios (Figure 1E). This offers the advantage of delivering an adequate drug dose while minimizing the required amount of carrier material.

The 2:2 ratio strikes the best balance among particle size, uniformity, stability, encapsulation efficiency and drug loading capacity. This makes it the optimal formulation for effective and reliable drug delivery, ensuring both stability during transit and therapeutic efficiency at the target site.

### 3.2. Critical Micelle Concentration (CMC)

**Figure 2:**
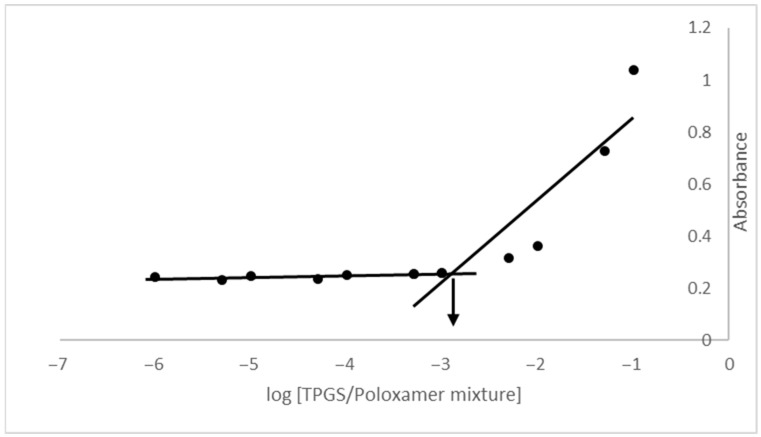
Plot of absorbance versus logarithmic of TPGS/Poloxamer 407 mixture concentration.

Critical micelle concentration (CMC) refers to the minimum concentration of an amphiphilic molecule required for micelle formation. From the graph of absorbance versus TPGS/Poloxamer mixture concentration in Figure 2, the CMC value is determined as 0.00125% *w*/*v*, which aligns with previous studies reporting CMC value for TPGS/Poloxamer 407 mixture in the range of 0.001–0.0015% *w*/*v* [36,37,38]. A low CMC value enhances the thermodynamic stability of TPGS/Poloxamer 407 micelle, allowing it to remain stable and maintain its integrity even when diluted in systemic circulation. This minimizes the risk of premature drug release during transit, thereby facilitating efficient drug delivery and controlled release to the target site.

### 3.3. Fourier-Transform Infrared Spectroscopy (FTIR)

**Figure 3:**
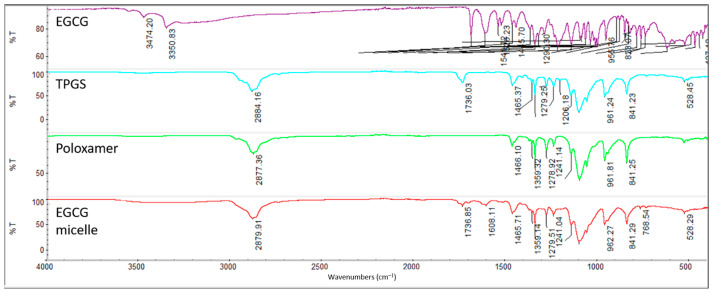
FTIR spectrum of EGCG, TPGS, poloxamer 407 and EGCG micelle.

The Fourier-transform infrared (FTIR) spectra of EGCG, TPGS, poloxamer 407 and EGCG micelle are analyzed to identify their characteristic functional groups (Figure 3). For EGCG, the O-H stretching of the phenolic hydroxyl group is observed at 3474.20 cm^−1^ and 3350.83 cm^−1^, while the C=O stretching of the gallic acid moiety appears at 1689.73 cm^−1^ and 1613.61 cm^−1^ [45,46,47]. In the spectrum of TPGS, the C-H alkane stretching vibration is detected at 2884.16 cm^−1^, the C=O stretching vibration at 1736.03 cm^−1^, the -CH_2_- bending at 1465.37 cm^−1^, the O-H bending at 1341.79 cm^−1^, and the C-O stretching at 1103.73 cm^−1^ [36,48]. Similarly, for poloxamer 407, the C-H alkane stretching vibration is recorded at 2877.36 cm^−1^, the -CH_2_- bending at 1466.10 cm^−1^, the O-H bending at 1341.59 cm^−1^, and the C-O stretching at 1099.61 cm^−1^ [36,49]. These characteristic peaks confirm the presence of the expected functional groups in the respective compounds. The similar peaks observed in the spectrum of EGCG micelle suggests that there is no significant interaction between EGCG and the excipients (TPGS and poloxamer 407).

### 3.4. X-Ray Diffractometry (XRD)

**Figure 4:**
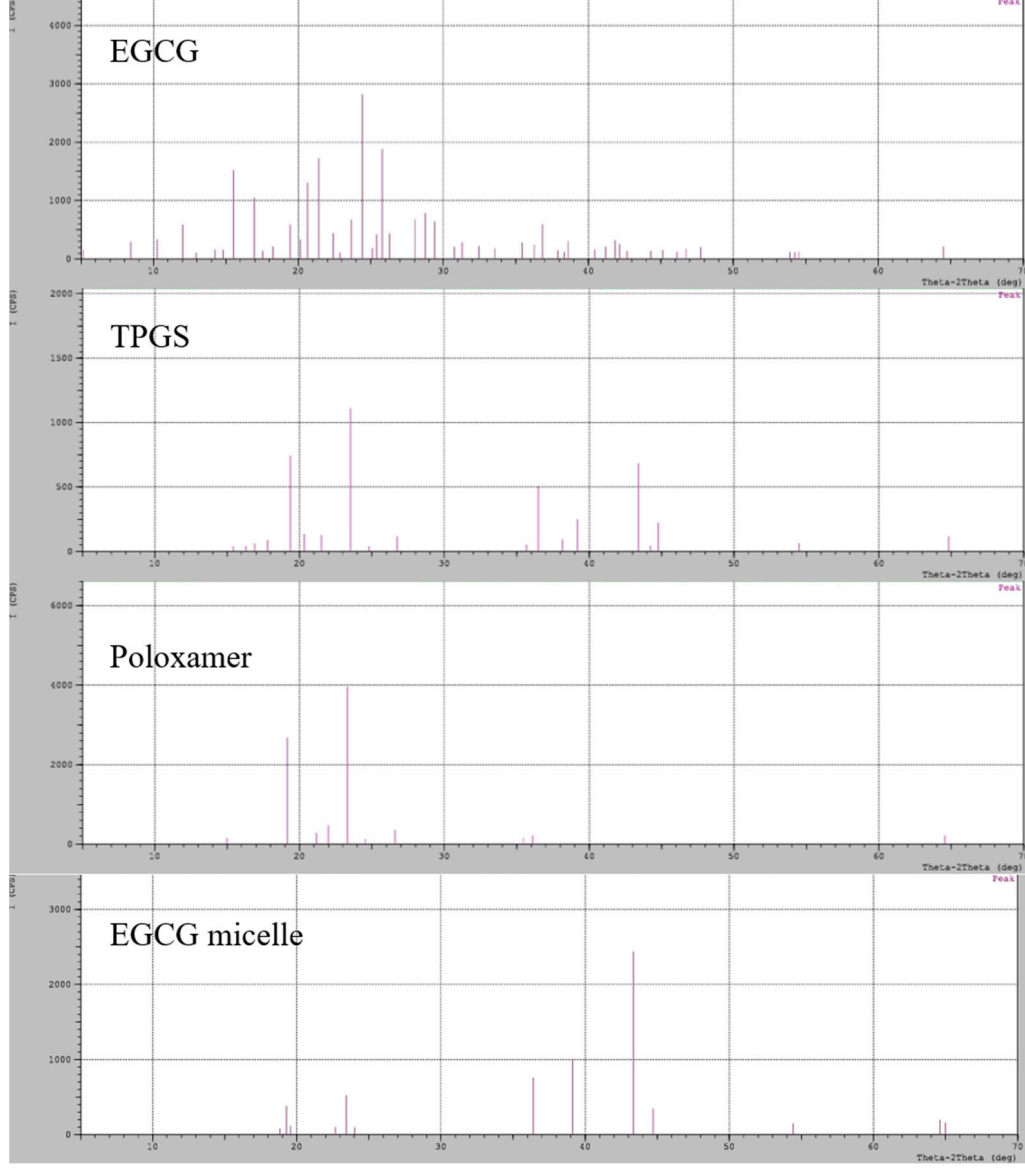
XRD pattern of EGCG, TPGS, poloxamer 407 and EGCG micelle.

The XRD analysis reveals that EGCG exhibits characteristic peaks at 2θ values of 21°, 24°, and 25°, confirming its highly crystalline nature (Figure 4). Similarly, TPGS and poloxamer 407 display strong crystalline peaks at 2θ values of 19° and 23°, indicating their well-ordered molecular structures. The similar diffraction patterns can be observed in other study [50]. In contrast, the EGCG micelle exhibits distinct peaks at 36°, 39°, and 43°, reflecting the structural transformation associated with micellization. Notably, the peaks at 19° and 23° are still present in the EGCG micelle but with significantly reduced intensity, suggesting a partial loss of crystallinity for TPGS and poloxamer 407 upon incorporation into the micelle. Interestingly, no characteristic peaks of EGCG are observed in the micelle, which strongly suggests that EGCG has been incorporated in the micelle in an amorphous state. This transformation into an amorphous form likely enhances EGCG’s dissolution and bioavailability, making it more suitable for biomedical applications.

### 3.5. Transmission Electron Microscopy (TEM)

**Figure 5:**
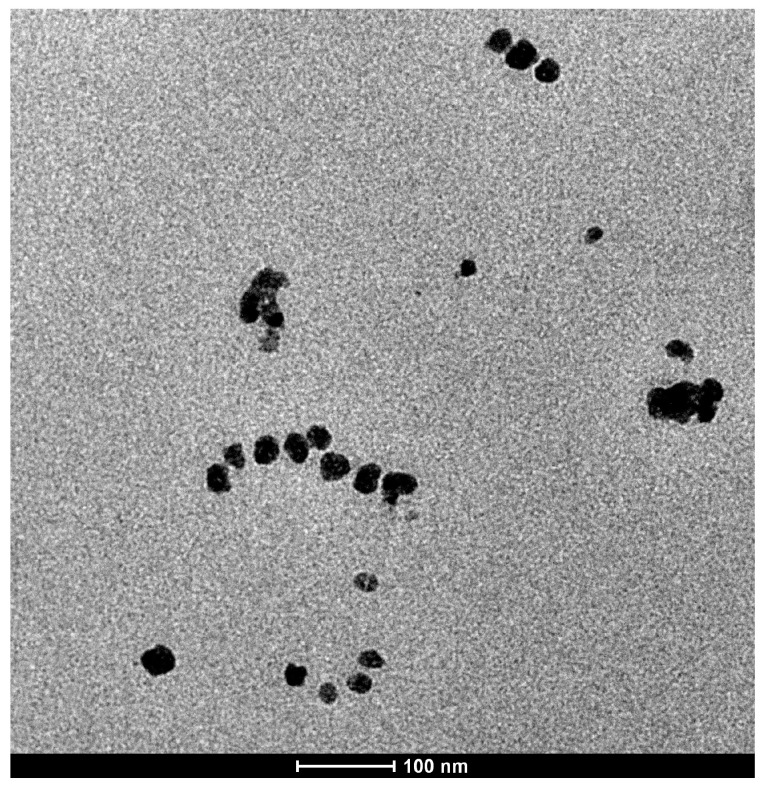
Representative TEM image of EGCG micelles.

The TEM image reveals that the particles are spherical in shape with uniform morphology (Figure 5). The size measured from the TEM image aligns closely with the data obtained using the DLS method, confirming the consistency and reliability of the results. While TEM provides a direct visualization of the particle structure at the nanoscale, DLS measures the hydrodynamic diameter, which includes the particle and any associated hydration layer. The agreement between these two methods indicates that the micelle formulation has a well-defined and stable size distribution, further validating the uniformity of the particles in the sample.

### 3.6. Storage Stability

**Figure 6:**
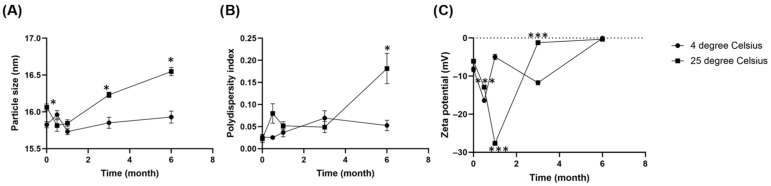
Changes in (**A**) Particle size (**B**) Polydispersity index (PDI) and (**C**) Zeta potential of freeze-dried EGCG micelle powder over 6 months of storage at 4 °C and 25 °C. Parameters were measured in six replicates and presented as mean ± SEM. The particle size and PDI of freeze-dried EGCG micelle remains at both 4 °C and 25 °C up to 3 months, while the value spikes at 6 months upon storage at 25 °C. Freeze-dried EGCG micelle stored at 4 °C maintains physical stability despite a gradual loss of zeta potential, while micelle at 25 °C shows a more pronounced and direct correlation between the loss of zeta potential and significant particle aggregation over six months. * *p* < 0.05 compared to 4 degree Celsius. *** *p* < 0.001 compared to 4 degree Celsius.

The particle size, PDI, and zeta potential of the freeze-dried EGCG-loaded micellar powders stored at 4 °C and 25 °C are shown in Figure 6. At 4 °C, the particle size shows minimal fluctuations, consistently staying below 16 nm (Figure 6A), and the PDI remains below 0.1 (Figure 6B), indicating the high uniformity of micelles. In contrast, a dramatic increase in both particle size and PDI becomes apparent after 3 months storage at 25 °C, with values spiking considerably by the 6-month mark. For micelles stored at 4 °C, despite the excellent physical stability in terms of size and homogeneity, the zeta potential exhibits concerning fluctuations, ultimately shifting significantly towards 0 mV by the 6-month mark (Figure 6C). This discrepancy suggests that while refrigerated conditions effectively slows down the physical aggregation process, the underlying electrostatic stability, which governs particle repulsion, is gradually diminishing. Time-dependent degradation is more pronounced and consistent across all parameters for micelles stored at 25 °C. The zeta potential experiences a drastic shift from adequately negative value towards 0 mV from 3 months onwards, indicating a complete loss of electrostatic repulsion, which correlates with the observed significant increase in particle size and PDI at 6 months.

Collectively, these results indicate that storage at 4 °C is more favorable for maintaining the micellar integrity and stability of the EGCG formulation for at least 6 months. Nevertheless, the declining zeta potential at 4 °C hints at a potential long-term electrostatic instability that warrants further investigation, possibly through extended storage studies or the exploration of cryopreservation techniques.

### 3.7. In Vitro Drug Release Study

**Figure 7:**
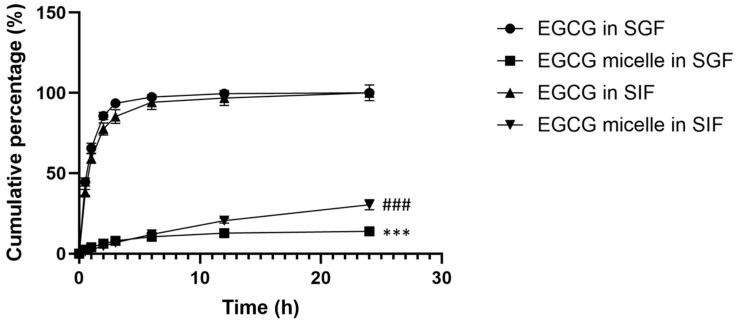
Cumulative release percentage of EGCG and EGCG micelle in simulated gastric fluid (SGF) and simulated intestinal fluid (SIF). This assay was carried out in triplicate, and results are presented as mean ± SEM. EGCG release from micelle is slower compared to free EGCG. *** *p* < 0.001 compared to EGCG in SGF at all time points. ### *p* < 0.001 compared to EGCG in SIF at all time points.

Figure 7 depicts the cumulative release percentages of EGCG and EGCG micelle in SGF over time. The release of EGCG is rapid, reaching nearly 100% within six hours. This suggests that EGCG, in its free form, dissolves quickly in the acidic environment of SGF. After the initial rapid release, the cumulative percentage plateaus, indicating that the dissolution of all available EGCG is complete within a short time. While the release of EGCG from micelle is much slower compared to free EGCG, with a cumulative release that gradually increases over time, suggesting that the micellar encapsulation effectively slows the release of EGCG in SGF. This controlled release is likely due to the protective structure of the micelles, which prevents the immediate release of EGCG in the acidic gastric environment. The free EGCG is rapidly released, making it more susceptible to degradation in the acidic gastric conditions, which reduces its bioavailability. In contrast, the slower release of EGCG from micelle demonstrates the benefit of encapsulation, as it offers sustained release and potentially enhances stability and bioavailability.

In SIF, the release profile for free EGCG shows a steep increase within the first few hours, indicating a burst release (Figure 7). Similarly, this rapid release can be attributed to the immediate solubility of free EGCG in the SIF. The release reaches a plateau at approximately 100% within the first 12 h, suggesting that most of the EGCG is fully dissolved and released into the medium during this period. The release profile for EGCG micelle shows a gradual and controlled release over 24 h. This indicates that the micellar encapsulation effectively slows down the release of EGCG, likely due to the diffusion or degradation of micelle over time. Even after 24 h, the cumulative release of EGCG from the micelles remains significantly lower than that of free EGCG, reaching around 30–40%. This controlled release could enhance stability and prolong the bioavailability of EGCG.

The release profiles of EGCG micelle in simulated gastric fluid and simulated intestinal fluid are analyzed using different mathematical models (Table S1). For EGCG micelle release in SGF, the Korsmeyer-Peppas model fits the data best, with an R^2^ value of 0.9985, indicating that EGCG micelle release in SGF follows a diffusion-controlled mechanism. On the other hand, the Korsmeyer-Peppas model provides the best fit for EGCG micelle release in SGF and SIF with the R^2^ value of 0.9985 ± 0.001 and 0.9998 ± 0.000, respectively. The n value for EGCG micelle in SGF falls between 0.43 and 0.85, suggesting an anomalous transport mechanism, where drug release is influenced by both classical diffusion and the relaxation of the micellar membrane. While the n value for EGCG micelle in SIF is 0.892, which approaches the threshold for Case II transport, where polymer chain relaxation plays a dominant role in drug release.

### 3.8. Preliminary Tolerability Observations

**Figure 8:**
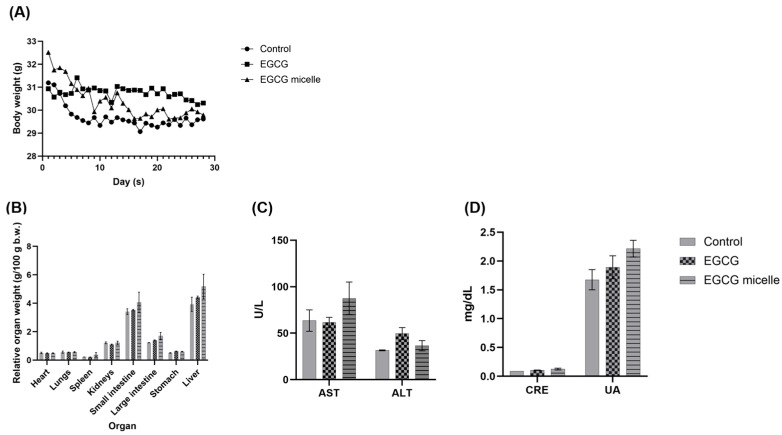
(**A**) Body weight (**B**) Organ index (**C**) Liver function profile and (**D**) Kidney function profile of control, EGCG, and EGCG micelle-treated groups over 28 days (*n* = 2, mean ± SEM). No overt adverse events were observed. AST: Aspartate Aminotransferase, ALT: Alanine Aminotransferase, CRE: Creatinine; UA: Uric Acid.

All animals survived, with only minor weight changes (<10%) observed, which remained within acceptable limits (Figure 8A). Cage-side observation revealed no overt adverse events were detected, suggesting good tolerability at the tested dose, in addition to comparable plasma AST, ALT, CRE and URE levels (Figure 8B–D). EGCG itself has an extensively characterized safety profile, with multiple studies [51,52,53,54,55], including our own prior work, demonstrating a NOAEL of 250 mg/kg b.w. in rodents [1]. In addition, another study reported that dietary administration of EGCG preparations to rats for 13 weeks was not associated with toxicity at doses up to 500 mg/kg/day [53]. Therefore, overt toxicity attributable to EGCG at the tested dose (100 mg/kg b.w.) was not anticipated. Moreover, both TPGS and poloxamer are FDA-approved excipients, recognized for their established safety, biocompatibility, and widespread use in pharmaceutical formulations, which further supports their suitability for this micellar system [33,35]. Accordingly, this study was designed to obtain preliminary observations on the tolerability of the EGCG loaded TPGS/poloxamer complex. Nevertheless, we acknowledge that the small group size inherently restricts statistical power, reduces generalizability, and may not capture rare or subtle toxicities. Therefore, these findings should be regarded as descriptive tolerability observations and not as a definitive toxicological evaluation. Larger studies with adequate sample sizes, inclusion of both sexes and extended endpoints are required to establish a comprehensive toxicity profile.

### 3.9. In Vivo Pharmacokinetics

**Figure 9:**
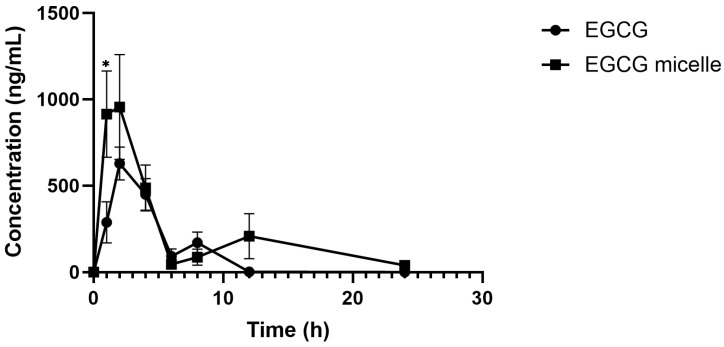
Plasma concentration versus time profiles of EGCG and EGCG micelle (*n* = 6–7, mean ± SEM). EGCG micelle demonstrates greater AUC and C_max_ than EGCG. * *p* < 0.05 compared to EGCG.

The plasma concentration–time curves of EGCG and EGCG micelle are presented in Figure 9, and the corresponding pharmacokinetic parameters are summarized in Table 2. The pharmacokinetic profile of EGCG is significantly improved upon micelle formulation, as evidenced by enhanced systemic exposure and prolonged circulation time. The area under the plasma concentration–time curve (AUC), a measure of overall drug exposure, increases markedly from 2758.03 ng/mL·h for free EGCG to 6277.62 ng/mL·h for the EGCG micelle, indicating improved bioavailability. Correspondingly, the peak plasma concentration (C_max_) is nearly doubled with the micelle formulation (1202.24 ng/mL) compared to free EGCG (655.70 ng/mL), suggesting enhanced absorption. Although the time to reach peak concentration (T_max_) is slightly delayed for the micelle (2.00 h vs. 1.71 h), this may reflect a more sustained release profile. Notably, the elimination half-life (T_1/2_) of EGCG is substantially extended from 1.53 h to 6.51 h in the micellar form, indicating a slower clearance and longer systemic retention.

The TPGS/poloxamer micelle formulation produced approximately a two-fold increase in Cmax compared with free EGCG. This enhancement can be explained by several corresponding mechanisms. First, micellar encapsulation protects it from oxidative and enzymatic degradation in the gastrointestinal tract, thereby increasing the fraction available for absorption. Second, micelles can promote intestinal uptake by facilitating both transcellular and paracellular transport, leading to more efficient permeation across the epithelial barrier. Third, the formulation may reduce immediate presystemic metabolism within the gut wall, allowing a greater proportion of intact EGCG to reach systemic circulation. These effects contribute to the higher peak plasma concentration observed with the micelle, consistent with previous reports demonstrating that TPGS nanocarrier systems can enhance the pharmacokinetic profile of EGCG despite its inherently low oral bioavailability [33,56].

The biphasic plasma concentration–time curves observed for both EGCG and EGCG micelles, with two distinct peaks, are in agreement with prior reports describing a similar pharmacokinetic pattern for EGCG [57]. The initial peak corresponds to direct intestinal absorption, whereas the second peak is indicative of enterohepatic recirculation, where conjugated EGCG metabolites excreted into bile are hydrolyzed in the intestine and subsequently reabsorbed as free EGCG. Compared with free EGCG, the micelle formulation exhibited a higher C_max_, a more pronounced secondary peak, and prolonged systemic exposure. These findings suggest that the micelle not only enhanced intestinal absorption but also facilitated reabsorption during enterohepatic recycling, thereby improving overall bioavailability.

Collectively, these findings indicate that micelle formulation enhances the bioavailability and systemic exposure of EGCG by reducing clearance and extending its residence time in circulation. However, it should be noted that the present pharmacokinetic evaluation was limited to single-dose administration, which may not fully capture drug disposition under repeated dosing conditions. While key parameters such as half-life and clearance provide a basis for estimating steady-state exposure and the likelihood of accumulation, definitive confirmation requires multiple-dose studies. Future investigations incorporating repeated-dose pharmacokinetics and tissue distribution analyses will therefore be essential to establish the long-term disposition and therapeutic potential of EGCG micelles.

## 4. Conclusions and Recommendations

In summary, the EGCG-loaded TPGS/Poloxamer 407 micelles developed in this study demonstrated favorable physicochemical characteristics, sustained drug release, and good storage stability and were well tolerated in a small-scale preliminary assessment following repeated oral administration. Importantly, the micelle system significantly enhanced the oral bioavailability of EGCG, as evidenced by the 2.27-fold increase in AUC and nearly two-fold rise in Cmax compared to free EGCG. The micelles exhibited nanoscale size (15.4 nm, PDI 0.16, zeta potential –17.7 mV), high encapsulation efficiency (82.7%), and adequate drug loading (7.6%). They displayed sustained in vitro release in both SGF and SIF, and freeze-dried powder remained stable for at least six months at 4 °C. The tolerability assessment was limited by the small sample size. Thus, the safety results should be interpreted with caution, and further investigations with larger cohorts, including both sexes and recovery groups, are warranted. Collectively, these results highlight the potential of this micellar system as a promising oral delivery platform for EGCG and related bioactives. Nevertheless, significant challenges remain, including the lack of scalable manufacturing processes, limited comprehensive toxicity and biocompatibility evaluations, and the absence of streamlined regulatory frameworks. These gaps must be addressed to ensure reliable development and safe translation of nanocarrier systems into clinical practice.

## Figures and Tables

**Table 1:** Cage-side observation criteria.

Observations	Category	Explanation
General condition	Normal	Awake, active, reacts to stimulation
	Mild	Burrows in litter, hides, lies still but is startled when handled
	Severe	Immobile, little or no voluntary movement. Burrows/hides. Presses head against cage bottom. Vocalizes. Extremely afraid and/or aggressive when handled
Porphyrin staining and/or eye inflammation	Absent	No discoloration, clean and clear eyes
	Mild	Some porphyrin and/or discharge around eyes and nose
	Severe	Obvious porphyrin on ‘face’ and/or on legs and paws. Eye(s) closed, squints and/or discharge around eye(s)
Movements and posture	Normal	Normal coordination without any difficulty in movements
	Mild	Moderate in-coordination when animal is stimulated; hunched posture
	Severe	Marked in-coordination, head held at angle, hunched posture and/or back, does not support itself on all four limbs and/or paralysis
Piloerection	Absent	Fur smooth and well-groomed
	Mild	Moderate piloerection
	Severe	Severe piloerection, sticky and poorly groomed fur
Skin	Normal	Skin covered entirely with fur. No sores or other signs of injury
	Mild	Small sores or scabs, no infection; scratching (signs of itching)
	Severe	Bites or scratches itself or trauma from others. Signs of infection such as redness and/or pus or serious discharge; sticky and poorly groomed fur. Non-healing operation wounds or broken sutures
Appetite/Food and water intake	Normal	Normal appetite, eating and drinking regularly
	Mild	Reduced appetite, consume less food and water
	Severe	No interest in food and appears dehydrated
Defecation	Normal	Firm fecal boli with brown colour
	Mild	Feces looser or harder than normal and/or abnormal colour
	Severe	Diarrhea (excessive watery stool)/Constipation (no stool or very hard stool) and/or abnormal colour
Urination	Normal	Normal urine colour (pale yellow to yellowish) without any odour
	Abnormal	Abnormal urine colour and/or has strong odour
Breathing Difficulties	Absent	Normal respiration, not strained or wheezy
	Present	Breathes with open mouth, abdominal breathing or panting, crackle and/or gasping noises

**Table 2:** Pharmacokinetic parameters of EGCG and EGCG micelle.

	Mean ± SEM	
Parameter	EGCG	EGCG Micelle
AUC (ng/mL·h)	2758.03 ± 463.91	6277.62 ± 1178.96 *
C_max_ (ng/mL)	655.70 ± 95.52	1202.24 ± 212.58 *
T_max_ (h)	1.71 ± 0.18	2.00 ± 0.45
T_1/2_ (h)	1.53 ± 0.16	6.51 ± 2.43

* *p* < 0.05 compared to EGCG. AUC: Area under curve, C_max_: Peak plasma concentration, T_max_: Time to reach peak concentration, T_1/2_: Half-life.

## Data Availability

The datasets used and/or analyzed during the current study are available from the corresponding author upon request.

## Supplementary Materials

The following supporting information can be downloaded at https://www.mdpi.com/article/10.3390/pharmaceutics17111441/s1. Table S1: Comparison of mathematical models for EGCG and EGCG micelle release in simulated gastric and intestinal fluids.
