# Supplementary material for: Development and Characterization of EGCG-Loaded TPGS/Poloxamer 407 Micelles with Evaluation of In Vitro Drug Release and In Vivo Pharmacokinetics and Tolerability Observations Following Oral Administration
Source: Pharmaceutics. 2025 Nov 7;17(11):1441. doi: 10.3390/pharmaceutics17111441 (PMC12655449; doi:10.3390/pharmaceutics17111441)
Supplement: Supplementary file 1 [file pharmaceutics-17-01441-s001.zip › pharmaceutics-3865420-supplementary.pdf]

SUPPLEMENTARY MATERIALS

Table S1. Comparison of mathematical models for EGCG and EGCG micelle release in simulated gastric and intestinal fluids

**Mathematical model**		**Simulated gastric fluid (SGF)**		**Simulated intestinal fluid (SIF)**	
		EGCG	EGCG micelle	EGCG	EGCG micelle
Zero-order	k_0	38.970 ± 0.711	2.950 ± 0.462	40.734 ± 0.622	2.368 ± 0.075
	R^2	0.6566 ± 0.049	0.9097 ± 0.015	0.6943 ± 0.004	0.9943 ± 0.002
First-order	k_1	1.154 ± 0.094	0.031 ± 0.005	1.230 ± 0.045	0.024 ± 0.001
	R^2	0.9981 ± 0.001	0.9196 ± 0.015	0.9971 ± 0.002	0.9959 ± 0.002
Korsmeyer-Peppas	k_{KP}	64.637 ± 2.581	4.049 ± 0.568	66.292 ± 1.107	2.599 ± 0.023
	n	0.392 ± 0.030	0.622 ± 0.022	0.415 ± 0.003	0.892 ± 0.029
	R^2	0.9925 ± 0.002	0.9985 ± 0.001	0.9907 ± 0.001	0.9998 ± 0.000
Hixson-Crowell	k_{HC}	0.316 ± 0.027	0.010 ± 0.002	0.334 ± 0.011	0.008 ± 0.000
	R^2	0.9855 ± 0.003	0.9164 ± 0.015	0.9948 ± 0.000	0.9954 ± 0.002
Higuchi	k_H	60.178 ± 1.336	4.417 ± 0.681	62.698 ± 0.967	3.449 ± 0.100
	R^2	0.9741 ± 0.011	0.9847 ± 0.004	0.9815 ± 0.001	0.9106 ± 0.009

Mathematical modeling of drug release. The release data obtained from the Section 2.11 was fitted to several mathematical models, i.e., zero-order model, first-order model, Korsmeyer–Peppas model, Hixson–Crowell model, and Higuchi model. Mt is the cumulative amount of drug release at time point t; M∞ is the total amount of drug loaded; t represent time; k_0, k_1, k_{KP}, k_{HC}, k_H represent the zero-order, first-order, Korsmeyer-Peppas, Hixson-Crowell, and Higuchi rate constants, respectively; and *n* represents the release exponent.
